# Revisiting early-stage COVID-19 strategy options

**DOI:** 10.12688/f1000research.23524.3

**Published:** 2021-04-23

**Authors:** Philip Machanick

**Affiliations:** 1 Computer Science, Rhodes University, Makhanda, Eastern Cape, 6140, South Africa

**Keywords:** COVID-19, early-stage pandemic intervention, asymptomatic transmission

## Abstract

**Background**: Early-stage interventions in a potential pandemic are important to understand as they can make the difference between runaway exponential growth that is hard to turn back and stopping the spread before it gets that far. COVID19 is an interesting case study because there have been very different outcomes in different localities. These variations are best studied after the fact if precision is the goal; while a pandemic is still unfolding less precise analysis is of value in attempting to guide localities to learn lessons of those that preceded them.

**Methods**: I examine two factors that could differentiate strategy: asymptomatic spread and the risks of basing strategy on untested claims, such as potential protective value of the Bacillus Calmette-Guerin (BCG) tuberculosis vaccine.

**Results**: Differences in disease progression as well as the possibility of alternative strategies to prevent COVID-19 from entering the runaway phase or damping it down later can be elucidated by a study of asymptomatic infection. An early study to demonstrate not only what fraction are asymptomatic but how contagious they are would have informed policy on nonpharmaceutical interventions but could still be of value to understand containment during vaccine roll out.

**Conclusions**: When a COVID-19 outbreak is at a level that makes accurate trace-and test possible, investigation of asymptomatic transmission is viable and should be attempted to enhance understanding of spread and variability in the disease as well as policy options for slowing the spread. Understanding mild cases could shed light on the disease in the longer term, including whether vaccines prevent contagiousness.

## Introduction

When I first submitted this paper in May 2020, I wrote with some urgency because I believed the issue of asymptomatic transmission of COVID-19 was not receiving the attention it deserved, and updated the paper before any reviews were in. All the versions of the paper, read in conjunction of the reviews, form a living review, showing progress in my understanding of the issue and progress in research into asymptomatic transmission.

As the COVID-19 pandemic has spread, its outcomes have differed by locality. In some, it has been contained quickly. In others, the rapid growth has slowed but not stopped. In many, the rapid growth has driven health systems to the point of collapse. New York state is the epicenter of the United States epidemic. What adds urgency to the search for alternative containment strategies is the fact that mortality rate (deaths as a fraction of population) in New York state on 22 April 2020 surpassed 1000 per million. If scaled to the entire country, this would be over 300,000 deaths ^1^. At time of writing the second version of this paper, US deaths were at almost 180,000 and that count exceeded 500,000 by the third version, illustrating that there is still work to be done at containment.

Since the pandemic is still playing out it is useful to reflect on the positive and negative outcomes and to try to map a way ahead for localities where it has not gone past the stage where it can easily be contained. Given that no known remedy exists for the disease, and that it is spreading too fast to rely on a vaccine to avoid major health or economic problems, non-pharmaceutical interventions (NPI) are the most critical thing to get right. Now that the pandemic has progressed beyond its initial stages, understanding asymptomatic transmission remains important as the question of universal mask wearing remains controversial ^1^ with inadequate testing of informal masks nonetheless at least justifying their use in terms of the precautionary principle ^2^. One study shows that viral shedding starts 2 days or more before symptoms show and that infectiousness peaks 1–2 days prior to symptoms and presymptomatic transmission is 37% to 48% and that this figure can be as high as 62% without adequate case finding. There are no definitive studies of asymptomatic transmission, despite evidence that it is real ^3^.

While understanding asymptomatic transmission alone does not fill the gap in investigating the efficacy of informal masks, it strengthens the case for applying the precautionary principle pending such a study. Studying informal masks is inherently difficult as there is so much variability, which is why I propose an initial focus on asymptomatic infection. Asymptomatic infection can also assist with understanding the highly variable progression of the disease. At a later stage of the pandemic, understanding transmission from milder cases could also be of value in understanding whether vaccines prevent further transmission.

A number of NPIs have been tried from social distancing to complete lockdowns. The consequence of acting too slowly is the risk of crashing a health system, which has hit some of the best in the world (Italy for example has historically been near the top of the World Health Organization’s ranking ^4^; and the epicentre of the COVID-19 outbreak is in the north ^5^, which has Italy’s strongest health resources ^6^. Though the pandemic at time of writing is still developing, it is worth reviewing options for countries able to avoid runaway exponential growth.

By way of example, I looked at options for South Africa, which embarked on a 21-day lock down ^7^ that started at about the time when 1,000 cases were reported (midnight, 26 March 2020; subsequently extended by another two weeks to 30 April), in the first version of this paper. At this much later stage of progress of the pandemic, countries where cases are declining have the same opportunity to use spare testing capacity for a study I propose in this paper.

I examine case studies in other localities encompassing the variability in outcomes and assess likely contributing factors to this variance. Given the shortness of time to make decisions, I do not attempt to develop a rigorous model but rely on extracting meaning from these case studies.

In my opinion, ignoring asymptomatic spread is a major error; a relatively simple experiment in a country like South Africa at the early stage of spread (or now at a later stage as outlined above) could validate this opinion. If proved correct, many lives could be saved. If proved incorrect, the cost is low relative to the benefit. I therefore urge that the experiment be carried out as a matter of urgency.

It is natural at an early stage of a rapidly-expanding pandemic to focus on the most serious cases as these are the ones where interventions make the biggest difference. Now that there is more time to assess evidence, there is also a case to do detailed studies of less serious cases to understand better what predicts progression to the worst effects. Identifying asymptomatic cases in particular could aid with this as they represent the extreme of the mild form of the disease.

In the remainder of the paper, I describe significant unknowns, work through cases studies and examine other factors, leading to the conclusion that a study of asymptomatic spread was the most urgent gap in early knowledge that should have been addressed to assess options for containment containment – and could still be of use at later stages of the pandemic. I propose a strategy for identifying and investigating asymptomatic cases.

## Significant unknowns

Because testing started under pressure, standards are not consistent ^8^. That means statistics like case fatality rate are problematic to compare across localities.

Another big unknown is the true number of infections since many that were not serious enough to require hospitalization may have resolved without being counted where testing was inadequate; if the asymptomatic fraction is as high as claimed in some instances, that also skews the case fatality rate high.

All of these factors also result in difficulty in establishing an accurate value for the basic reproduction rate *R*_0_, the initial mean number of infections per infected case. The value of *R*_0_ matters for computing the herd immunity level, widely reported in the mass media as 60% of the population, the number used to justify the initial British response of allowing it to run through the population ^9^. As infections spread, the effective reproduction number at time *t*, *R _t_*, will decline below 1, the herd immunity threshold ^10^. NPIs artificially force *R _t_* to drop, faking the effect of a less contagious disease. However, if NPIs are relaxed before herd immunity is reached, another round of rapid increase can ensue as *R _t_* again rises above 1. It is for this reason that estimation of *R _t_* is useful ^11^.

Herd immunity occurs when the fraction of the population immunised (either by vaccination or by acquiring immunity post-infection) exceeds the threshold *P _herd_* in Equation 1,

$$P_{herd}=\frac{1}{E}\left(1-\frac{1}{R_{0}}\right)\;(1)$$

where *P _herd_* is the fraction of the population at which infections peak ( *R _t_*=1) and *E* is the effectiveness of immunisation. If immunisation is 100% effective ( *E* = 1), Equation 1 becomes ^12^:

$$P_{herd}=1-\frac{1}{R_{0}}\;(2)$$

**Figure 1.**
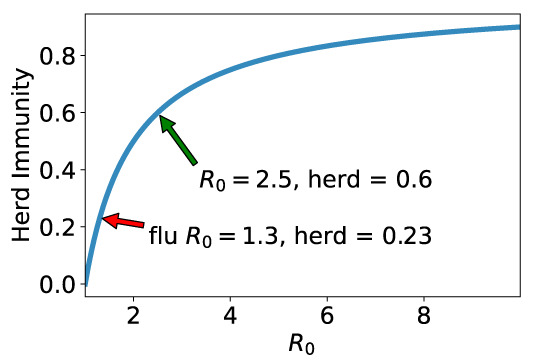
Herd immunity varying with basic reproduction rate *R*_0_. If *R*_0_ for COVID-19 is 2.5, herd immunity occurs after 60% of the population is infected (green arrow). The mean *R*_0_ value for seasonal influenza results in herd immunity at about 23% of the population (red arrow).

Figure 1 illustrates how *P _herd_* varies with *R*_0_ (assuming *E* = 1; in the absence of a vaccine, this means that any recovered cases cannot be reinfected). For seasonal influenza, if *R*_0_ = 1.3, the herd immunity threshold is 23%. For *R*_0_ = 2.5, the herd immunity threshold is 60%. However, if the true COVID-19 *R*_0_ value is significantly higher, so is the the herd immunity threshold. For example, if *R*_0_ = 4, *P _herd_* is 75%.

Even for influenza, *R*_0_ can vary widely depending on the strain. For the H1N1 strain, *R*_0_ was estimated at 1.4–1.6; for the 1918 flu, the estimated *R*_0_ range is 1.4–2.8 and even seasonal flu has a wide *R*_0_ range of 0.9–2.1 ^13^.

One study reports COVID-19 *R*_0_ values varying from 1.4 to 3.8 ^14^. Another narrows the range to 2.24 to 3.58 ^15^.

**Figure 2.**
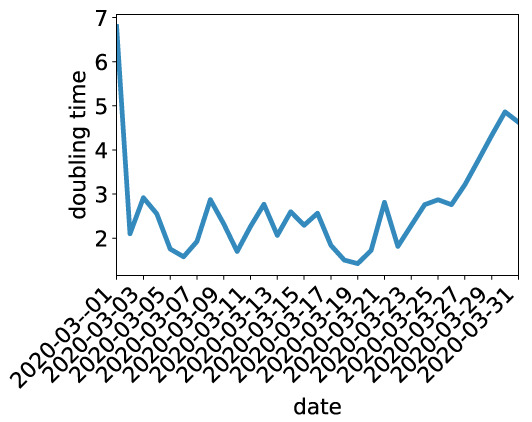
Doubling time in the US: 1–31 March 2020. For much of the month, doubling time is 2–3 days, implying that *R _t_* is not varying significantly.

A model with *R*_0_ = 2.68 yields a doubling time of 6.4 days ^16^. (which would hold good until the fraction susceptible dropped enough to reduce *R _t_*, unless NPIs artificially reduced *R _t_* – it is this phase of expontial growth that puts health systems under pressure). The doubling time was far shorter than this during peak growth in places where it was not under control. In the United States, for example, doubling time was less than 3 days 1–24 March 2020 (see Figure 2, based on the rule-of-70 ^17^). That rapidity of growth suggests that *R*_0_ is on the high rather than low side of published estimates, but the low level of testing in the USA at early stages of the pandemic make it difficult to derive robust measures.

With so much uncertainty, relying on herd immunity is folly, as was discovered in the UK ^9^.

Broadly speaking, countries with a tradition of personal liberty and rejecting authority have found it hard to adapt to NPIs like social distancing ^18^. Informal settlements and other high-density dwellings of the poor also make social distancing hard ^19^. For this reason, it is important to explore all alternatives including those that were missed in early stages so that other countries that are being hit later can learn the right lessons.

## Methods

I use Python 3.7.4 within the Jupyter Notebook environment version 6.0.3 to do anyalysis. All the code used is archived on GitHub ^20^ and is labelled as version 1.1.1.

Data is from the Worldometers web site and papers cited; data used in analysis is embedded in the GitHub archive ^20^. A snapshot of the Worldometers site containing the data used is archived at Webarchive (updated in Webarchive for Version 2 of the paper).

Herd immunity is calculated based on the standard formula assuming exponential growth using Equation 2.

I calculate doubling time for March 2020 using the rule-of-70, which assumes exponential growth and is accurate under that assumption:

$$t_{double}=\frac{70}{r_{growth}}\;(3)$$

Where the growth rate is expressed as a fraction of 100. For example, if the growth rate *r _growth_* = 40% then:

$$t_{double}=\frac{70}{40}=1.75\;(4)$$

The number 70 derives from the fact that 0.7 ≈ ln(2) ^17^.

## Case studies

I consider three cases: instances where the disease has run its course, examples where containment has slowed the growth to the point of being manageable and finally examples where growth has severely stressed health systems.

These examples are not meant to be exhaustive but provide archetypes of different types of outcome.

### Run to completion

I use two types of localities as examples of where the disease has effectively run to completion: cruise liners and a village in Italy, Vo’Euganeo. While localities like Taiwan and New Zealand may not have run to completion, they are useful to include as a retrospective on a potential elimination strategy.

I take Diamond Princess, a widely cited case, as representative of the first kind. 

### Cruise liner

Diamond Princess is one of the most documented examples. Of the 3,711 passengers and crew on board, 634 tested positive out of the 3,063 tested and 328 were asymptomatic ^21^ – meaning that over 50% were asymptomatic.

It may seem startling how few tested positive. Given the constrained environment of a cruise liner, a higher rate of social mixing would be expected than in normal living conditions. Once it was known that there was an infectious condition on board, effective NPIs could explain this and result in reducing *R _t_*.

However the major figure to take way from this is the fact that over half were asymptomatic.

### Italian village

For the Italian village, the number of cases that tested positive but were asymptomatic were in the range of 50–75% ^2^ ^22^. More recent meta-anslyses have suggested that the true asymptomatic fraction may be as low as 15–20% ^23– 25^. Classifying a case as asymptomatic may arise from not following up, since pre-symptomatic infectiousness can start several days before symptoms show ^26^.

What is particularly noteworthy is that 100% of the population was tested so no asymptomatic cases were missed. This completeness of coverage does not guarantee that a similar result would occur in a different population but at least provides a clear data point. All positive cases were isolated. The number who were ill fell from 88 to 7 in 10 days.

By contrast with this strategy, on 25 February 2020, the strategy for the rest of Italy changed from broad testing of all contacts to a focus on only testing those who had clear symptoms and required hospitalization ^27^. This decision was taken when the country had less than 100 cases.

It is a puzzle why it should be more important to test cases who must be hospitalized anyway rather than find those in the community who may be contagious.

I quote the advice of Italian immunologist Prof Romagnani (via Google Translate):

It is therefore absolutely essential to extend the swabs to the majority of the population, in particular to risk categories (i.e. exposed to multiple contacts), and therefore isolate the virus positive subjects and their contacts, even if asymptomatic, as early as possible. In particular, it is absolutely necessary to swab all those who have a high probability of transmitting the virus, especially if they live in closed communities with multiple and close contacts. Finally, it is very important that all those at risk wear masks, not so much to protect themselves from infection, but rather to protect others from themselves, even when they do not show symptoms.

Even if we take the lower figure of 50% of the Vo’Euganeo being asymptomatic, ignoring this factor in early testing strategies was a major error. However, in a larger population it is not practical to test everyone and not necessarily useful as a person tested negative could subsequently be infected. Instead, treating everyone exposed to a known case as infected until proved otherwise would catch a significant fraction of the asymptomatic cases.

### Elimination

Taiwan and New Zealand both had robust public health responses that effectively stopped the pandemic. In both examples, contact tracing and testing were followed by isolation of all close contacts and quarantining of positive cases. The main difference between the two is that Taiwan was ready at the outset, whereas New Zealand had to use lockdowns to bring infections under control. The key requirement for an elimination strategy is treating *all* close contacts as potentially infected, underlining the role of asymptomatic transmission ^28^.

### Slower growth

Several countries have managed to contain exponential growth after a major outbreak. Two examples are China and South Korea.

Since China had the first outbreak, they did not act fast enough and had to close down a major part of their economy. Much of Hubei province was effectively placed under quarantine on 23 January 2020, using methods such as tracking social media to enforce it. Since China is a large country, it was possible to isolate the infected region and rush in resources from the rest of the country. Overall the measures used seem unlikely to be transferable to most other countries ^29^.

South Korea instead took an approach of rapid and comprehensive testing while isolating all known cases *and all their contacts* ^30^. With no lockdown, they brought the increase off the exponential trend. That supports my opinion of how the Italian test strategy went wrong.

### Rapid growth

The United States only showed signs of breaking exponential growth after over 300,000 cases were reported on 4 April 2020. Some days during March 2020, growth was at 40% or more per day with a doubling time of two days and, up to 24 March, doubling time was generally three days or less.

Over the period 2–20 March 2020, growth in the US ranged from 24% to 49% per day. With that level of growth, the United States had no option but to implement increasingly country-wide lockdowns. Other countries that resisted this strategy ran into exactly the same issue: exponential growth defeated their health systems.

In a relatively large country like the US, if growth is not even across the country, there is the option to rush resources to the hotspots as was done in China. However, this option is not as attractive as stopping the spread much earlier as the hotspots become extremely resource intensive. New York state for example has been widely reported as estimating a requirement of 40,000 ventilators at peak ^3^ though the actual peak number of cases requiring ventilation did not come close to this level ^31^.

Localities that have suffered this sort of resource intensity have also generally had higher case fatality rates. This could in part be an artefact of inadequate testing that under-counts cases. However Italy (13% case fatality) has a *higher* per capita rate of testing than Germany (3.4% case fatality) as of 20 April 2020: 23,985 per million population *vs*. 20,629 for Germany. Under-counting asymptomatic cases is not likely to be the sole issue.

## Other factors

An important factor to consider is comorbidities. South Africa has high rates of tuberculosis and HIV infection, both of which are significant risk factors for any pulmonary disease ^32^. In one study in Italy, out of 355 patients who died, only 3 (0.8%) had no prior condition. 25.1% had one condition, 25.6% had two and 48.5% had three or more ^27^.

Balanced against this is early statistical evidence that differences in national coverage of the Bacillus Calmette-Guerin (BCG) tuberculosis vaccine explain differences in case fatality rates ^33^. This study is not peer reviewed and therefore should not be relied on too strongly. A pure statistical study without direct causal evidence points to the need to establish causality rather than signifying causality.

Weighed against relying on BCG coverage before further evidence emerged is that Iceland, which does not have mandatory BCG vaccination ^34^, was very successful in slowing the spread by an aggressive testing programme, including quarantining everyone who had contacted a known case ^35^.

More recent evidence since early versions of this paper justifies caution: there is at best mild evidence of a protective effort and no evidence that BCG slows the spread or reduces serious cases ^36^.

A broader lesson arises out of the early claims about BCG: a statistical study without causality could turn out to be coincidence. Another example is a study of invermectin in Peru that appears to show a close link between ivermectin use and reduced mortality ^37^. Yet one of the first reasonably rigorous peer-reviewed studies of ivermectin shows it has no significant therapeutic effect ^38^.

Finally there is indirect evidence of asymptomatic spread in the apparent efficacy of masks in slowing spread. While early WHO advice was against the asymptomatic mask wearing, this was part of an advisory that aimed to prevent a run on medical-grade masks ^39^. More recently there is support for cloth masks being worn by the public ^2^ and experimental evidence that surgical masks block aerosol transmission of coronaviruses and influenza viruses ^40^. While some still doubt the evidence, the fact that mandatory mask wearing was a factor in reducing the spread of the 1918 influenza pandemic ^41^ supports the case for encouraging mask-wearing as a protection for the COVID-19 pandemic provided that this does not deplete supplies of medical-grade masks and there is a programme of public education on use of masks and their role in the context of other measures like distancing and hygiene.

Given the high risk associated with comorbidities, it is premature to place too much reliance on any mitigating factors other than slowing transmission. NPIs are the main game until pharmaceutical options – including vaccines – become viable on a large scale.

## Proposed research

Since earlier versions of this paper, a systematic meta-analysis and review of viral load dynamics, shedding and infectiousness has shed some light on asymptomatic transmission, indicating that asymtomatic infection is contagious but not for as long as symptomatic infection ^42^. One study estimates the relative infectiousness of asymptomatically infected cases to be 0.27 (if on a small number of cases).

Since one of the biggest unknowns was the prevalence and contagiousness of asymptomatic infection, I proposed a project in earlier versions of this paper to identify such cases early and identify informative features. Features of interest include:

- viral shedding after initial infection; this should include variability in magnitude and duration
- prevalence in asymptomatic cases of comorbodities and risk factors like age
- testing for antibodies including those for related but more benign coronaviruses
- testing for T cell variability and other features of the immune system that may influence disease progression ^43^

To identify these cases in time to measure viral shedding from the start of infection, comprehensive contact tracing of a representative sample of the infected population is necessary. Any who test positive out of this cohort can be followed up by a further round of contact tracing; if this finds more positive cases that have been in contact with only asymptomatic cases, that would provide a measure of asymptomatic contagion. If however the programme prevents secondary infections, viral shedding will still be a useful indicator of contagiousness. In one study, rapid identification and isolation of asymptomatic cases prevented secondary infections ^11^ but this should not be taken as indicative that asymptomatic cases are not contagious.

This is a project that could be carried out with a modest burden on testing capacity – as long as the pandemic is not expanding at full pace.

Since the earlier versions of this paper, there have been some advances in understanding asymptomatic infection, including studies that show asymptomatic cases develop antibodies, if at a lower level than symptomatic cases ^44, 45^. However, given that we are no longer at the early stage of the pandemic, another related concern needs to be added to the mix: how contagious a vaccinated person could be if they are infected – despite not developing symptoms. I conjecture that this could follow the same pattern as asymptomatic infection without a vaccine: a lower rate or shorter duration of viral shedding, or both. An early study of the Pfizer–BioNTech vaccine (BNT162b2) supports this conjecture but more systematic studies over a range of vaccines will be useful to characterize the extent to which transmission is suppressed, as opposed to the primary endpoints of vaccine trials, reducing serious illness and death ^46^. There is considerable variability in primary endpoints in vaccine trials though the public health interest focuses on severe disease so vaccine trials mostly focus on symptomatic infection with various definitions of “severe” ^47^.

The last question I raise on T cell variability has been addressed – even asymptomatic cases who are anti-body seronegative have a robust SARS-CoV-2-specific T Cell response ^48^. This bodes well for vaccine efficacy.

## Conclusions

The most significant finding out of this review is that we do not know enough about asymptomatic transmission.

This is a problem easily remedied in early-stage spread – or at a later stage when cases are reducing – and there is spare test capacity.

The proposed project would give a clearer picture of the potential for asymptomatic spread and add to the evidence for universal mask wearing.

If the experiment to measure asymptomatic spread shows that it is a significant factor, that signifies a change in testing strategy. Should any asymptomatic positives be discovered, more aggressive action should follow: they and all their contacts should be quarantined and released when they no longer test positive, taking into account time for incubation.

South Africa, at the date of the first version of the paper, had less than 3500 cases; at time of writing the second version, the daily number of cases had declined to 25% of the peak. Going back to test all contacts was doable at the outset; with test demand 25% lower than at peak, testing all contacts of a representative sample is practical. While it is possible that despite a significant asymptomatic fraction, asymptomatic infection is not contagious, the cost of finding this out is very low compared with the cost of not containing the spread. Iceland does not do widespread BCG vaccination ^34^ and yet was successful in containing the spread. The Iceland experience indicates that there is no need to rely on questionable evidence such as that the BCG vaccine provides protection: act early and catch all cases *including the asymptomatic* and the spread can be curtailed ^35^. However, if we know exactly how contagious aymptomatic cases are, that will further inform NPI strategy.

The cost of taking the step I advocate is far lower than the cost of allowing asymptomatic spread to get out of control. If it leads to an effective containment strategy, it will also remove the need for an economically damaging extended lockdown. Even at a later stage of the pandemic, informing policy choices like mask wearing is useful, as is better understanding of the drivers in variabililty of the disease. And economically damaging NPIs can be better avoided if we understand the disease better – how it spreads, what makes people more vulnerable.

In the time since the first draft of this paper, studies have pointed to a lower fraction of asymptomatic transmission than earlier reports but the fraction is still high enough to be a significant factor in transmission. However, I am still of the view that studying asymptomatic transmission is important as a clue to what differentiates milder cases, and could also shed light on the extent to which vaccines stop transission, as opposed to restricting infections to casuing a milder illness.

As with the early stage of the pandemic, milder to asymptomatic cases are attracting less interest in vaccine trials because they do not impose a direct public health burden. However, I argue that they impose an indirect public health burden as long as they may be contagious.

## Data availability

### Source data

COVID-19 data used was gathered from Worldometer: https://www.worldometers.info/coronavirus/.

Data is archived at: http://web.archive.org/web/20200422135436/https://www.worldometers.info/coronavirus/ and the update for the second version is archived at http://web.archive.org/web/20200822095038/https://www.worldometers.info/coronavirus/.

### Extended data

The Python code used to graph herd immunity versus *R*_0_ as well graphing doubling time and the US exponential growth scenarios is available from GitHub: https://github.com/philip-mach/herd-immunity.

Zenodo: philip-mach/herd-immunity: includes herd immunity and doubling time graphs. http://doi.org/10.5281/zenodo.3762356 ^20^.

Data is available under the terms of the BSD 2-clause license.

## Footnotes

^1^The data source for cases and fatalities, unless otherwise stated, is the Worldometers web site.

^2^The wide range is not explained – possibly defining asymptomatic is imprecise.

^3^For example: https://abc7ny.com/ventilators-coronavirus-new-york-ny-cases-in/6086097/.
