# Supplementary material for: Revisiting early-stage COVID-19 strategy options
Source: F1000Res. 2021 Apr 23;9:327. Originally published 2020 May 4. [Version 3] doi: 10.12688/f1000research.23524.3 (PMC8127020; doi:10.12688/f1000research.23524.3)
Supplement: Peer review history

# Peer review: Reviewer response for version 3

**DOI:** 10.5256/f1000research.56040.r83832

**Published:** 2021-05-14

**Authors:** Siddharth Sridhar

**Affiliations:** 1 Department of Microbiology, University of Hong Kong, Hong Kong, Hong Kong

I approve the current version of the manuscript.

Is the work clearly and accurately presented and does it cite the current literature?

Partly

If applicable, is the statistical analysis and its interpretation appropriate?

Not applicable

Are all the source data underlying the results available to ensure full reproducibility?

Partly

Is the study design appropriate and is the work technically sound?

Yes

Are the conclusions drawn adequately supported by the results?

Partly

Are sufficient details of methods and analysis provided to allow replication by others?

Yes

Reviewer Expertise:

NA

I confirm that I have read this submission and believe that I have an appropriate level of expertise to confirm that it is of an acceptable scientific standard.

# Peer review: Reviewer response for version 3

**DOI:** 10.5256/f1000research.56040.r83833

**Published:** 2021-04-26

**Authors:** Agata Mikolajczyk

**Affiliations:** 1 Department of Biochemistry and Molecular Biology, Faculty of Veterinary Medicine, Wroclaw University of Environmental and Life Sciences, Wroclaw, Poland

I have no further comments for this article.

Is the work clearly and accurately presented and does it cite the current literature?

Yes

If applicable, is the statistical analysis and its interpretation appropriate?

Not applicable

Are all the source data underlying the results available to ensure full reproducibility?

Yes

Is the study design appropriate and is the work technically sound?

Yes

Are the conclusions drawn adequately supported by the results?

Partly

Are sufficient details of methods and analysis provided to allow replication by others?

Yes

Reviewer Expertise:

microbiology, molecular biology, translational medicine

I confirm that I have read this submission and believe that I have an appropriate level of expertise to confirm that it is of an acceptable scientific standard.

# Peer review: Reviewer response for version 2

**DOI:** 10.5256/f1000research.29077.r77927

**Published:** 2021-02-09

**Authors:** Agata Mikolajczyk

**Affiliations:** 1 Department of Biochemistry and Molecular Biology, Faculty of Veterinary Medicine, Wroclaw University of Environmental and Life Sciences, Wroclaw, Poland

1. Although author of this work arise few types of COIVD-19 course among lot of variety, it gives some important general view. Nevertheless there is no type where pandemic were managed very effectively. Only there is some discussion about Iceland giving an example of extensive testing but this could be support with showing strategy of other countries which managed the best the COVID - 19 like New Zeland or Taiwan and point what was the common for this three countries.
2. Because work was writing some time ago and situation during pandemic is changing very dynamically especially in the context of vaccines, some of given strategies how to managed with current pandemic can be not timely .  However it has general value as strategy for coping with any pandemic so in my opinion it is important content of this work.
3. There is lack of any detailed data which correlate wearing mask with spread of virus. There is some discussion about this and references, but here could be some investigation like: comparing spread rate of virus in countries after  obligated  society to wearing mask always with the one which hadn’t obligate.
4. As I could see the report from second reviewer I will not double the point he has raised and I support his comments.

Is the work clearly and accurately presented and does it cite the current literature?

Yes

If applicable, is the statistical analysis and its interpretation appropriate?

Not applicable

Are all the source data underlying the results available to ensure full reproducibility?

Yes

Is the study design appropriate and is the work technically sound?

Yes

Are the conclusions drawn adequately supported by the results?

Partly

Are sufficient details of methods and analysis provided to allow replication by others?

Yes

Reviewer Expertise:

microbiology, molecular biology, translational medicine

I confirm that I have read this submission and believe that I have an appropriate level of expertise to confirm that it is of an acceptable scientific standard, however I have significant reservations, as outlined above.

# Response

**Published:** 2021-02-09

**Authors:** Philip Machanick

**Affiliations:** Rhodes University, South Africa

Thank you for your constructive comments and being willing to accept that an article that has taken a long time to get through review is worth updating for the current context.

 When I first wrote it, I hoped to get it through review fast to add to the debate about asymptomatic transmission.

 When I had no reviews on the first version, I updated it; doing so again, thanks to the f1000 model of keeping all version live, unintentionally turns it into a living review.

# Response

**Published:** 2021-04-09

**Authors:** Philip Machanick

**Affiliations:** Rhodes University, South Africa

1. Although author of this work arise few types of COIVD-19 course among lot of variety, it gives some important general view. Nevertheless there is no type where pandemic were managed very effectively. Only there is some discussion about Iceland giving an example of extensive testing but this could be support with showing strategy of other countries which managed the best the COVID - 19 like New Zeland or Taiwan and point what was the common for this three countries.
  Thank you for raising this point. Now that we have more history, New Zealand and Taiwan present an interesting contrast in how to eliminate community spread – respectively by locking down hard and early before being ready for the appopriate measures versus having the measures in place already ^1^. I added this in to the case studies.
2. Because work was writing some time ago and situation during pandemic is changing very dynamically especially in the context of vaccines, some of given strategies how to managed with current pandemic can be not timely .  However it has general value as strategy for coping with any pandemic so in my opinion it is important content of this work.
  Thank you – I agree. As I was preparing my update, I heard the British Prime Minister, in response to a question on his biggest mistake, admitting that it was failing to consider asymptomatic transmission.
3. There is lack of any detailed data which correlate wearing mask with spread of virus. There is some discussion about this and references, but here could be some investigation like: comparing spread rate of virus in countries after  obligated  society to wearing mask always with the one which hadn’t obligate.
  Since the first reviewer pointed out that coverage of masking was inadequate I decided to remove this as a major focus. I have attempted to look into this issue but there are too many confounders to arrive at a definitive conclusion (e.g., if mask-wearing reduces other NPIs in some societies, that would reduce the effectiveness of masks) ^2^. In general terms the fact that asymptomatic transmission is a factor indicates that mask wearing is likely to be effective, but I have found no definitive study to back this – though e.g. the Czech experience of having one of the lowest rates of infection in Europe while masking and becoming one of the worst when masking was dropped supports the case. For this reason, I prefer to make the focus asymptomatic transmission and dropped masking as an issue.
4. As I could see the report from second reviewer I will not double the point he has raised and I support his comments.
  Thank you – see my responses to the other reviewer.

**References**

 1. Summers J, Cheng HY, Lin HH, Barnard LT, Kvalsvig A, Wilson N, Baker MG. Potential lessons from the Taiwan and New Zealand health responses to the COVID-19 pandemic. *The Lancet Regional Health-Western Pacific*. 2020 Oct 21:100044.

 2. Cartaud A, Quesque F, Coello Y. Wearing a face mask against Covid-19 results in a reduction of social distancing. Plos one. 2020 Dec 7;15(12):e0243023.

# Peer review: Reviewer response for version 2

**DOI:** 10.5256/f1000research.29077.r74339

**Published:** 2020-11-09

**Authors:** Siddharth Sridhar

**Affiliations:** 1 Department of Microbiology, University of Hong Kong, Hong Kong, Hong Kong

This article is a description on some knowledge gaps surrounding asymptomatic COVID-19 burden. Good primer on herd immunity thresholds in the introduction. Some nice case studies. Reasonable discussion on the importance of asymptomatic/ pre-symptomatic transmission. However, there are some areas that require attention.

 Overall, this manuscript reads more like a commentary/ narrative review rather than an original 'research article'. 

 My specific criticisms are as follows: 

1. Define variables in equation 1 below the equation. 
2. Apart from R _0_, the concept of the effective reproduction number should also be introduced as a much more meaningful figure when NPIs are enforced.
3. I recall closer to 700 cases on Diamond Princess. Please verify.
4. “What is startling about these numbers is how few tested positive. Given the constrained environment of a cruise liner, a higher rate of social mixing would be expected than in normal living conditions.” This is somewhat simplistic. Once the outbreak started, you would expect significant reduction in social interactions and increasing mask usage/ isolation among passengers on board the Diamond Princess, which would lower the R _t_.
5. I do not understand how figure 2 and the equations relate to the rest of the paper. In fact, the first paragraph of the methods appears unrelated to the narrative case study/ review in the rest of the manuscript. 
6. I agree with the author on the importance of detecting asymptomatic shedders, but most current estimates of true asymptomatic COVID-19 rates are less than 50% (15 – 30% according to some meta-analyses). This needs to be updated. There is also a need to differentiate between true asymptomatic infection and ‘pre-symptomatic’ cases who are detected before eventually going on to detect symptoms.
7. Consider citing Escobar LE *et al* ^1^, PNAS as a peer-reviewed reference on the impact of BCG vaccination on severe COVID-19.
8. BCG vaccination is unlikely to offer sterilizing immunity against COVID-19. Instead, it is mostly being investigated for its effect on ameliorating immune responses to prevent severe COVID-19. Therefore, we can’t rely on BCG vaccination to slow spread of COVID-19 and it is not a replacement for good-practice NPIs. Any country that practices high standards of NPIs, test-and-trace, and isolation-and-quarantine would be able to control COVID-19 irrespective of its BCG coverage. I feel the text is implying that BCG vaccination is under investigation for reducing COVID-19 community burden and this should be modified.
9. “Another possible mitigating factor is that existing HIV remedies are among those being investigated for efficacy against COVID-19. However, it would be foolhardy to rely on this as a mitigating factor without solid evidence that it applies widely enough to matter.” May delete this point as lopinavir/ritonavir has been proven to be of no benefit in hospitalized COVID-19 patients in the WHO SOLIDARITY trial.  
10. For the points in proposed research, several natural experiments looking at viral shedding in asymptomatic individuals (e.g. Han MS et al, Emerg Infect Dis ^2^) and infectivity of asymptomatic cases (Gao M et al, Respir Med ^3^) have already been described. Characterization of humoral and T-cell responses to COVID-19 have also been done in mild/ asymptomatic cases (Ko JH et al, J Clin Med ^4^). The author may consider updating this section and reviewing the literature thoroughly to identify current research gaps.
11. The methods section in the abstract promised an examination of the strategy of using cloth face masks. However, there is no substantive discussion of cloth face masks in the text.

Is the work clearly and accurately presented and does it cite the current literature?

Partly

If applicable, is the statistical analysis and its interpretation appropriate?

Not applicable

Are all the source data underlying the results available to ensure full reproducibility?

Partly

Is the study design appropriate and is the work technically sound?

Yes

Are the conclusions drawn adequately supported by the results?

Partly

Are sufficient details of methods and analysis provided to allow replication by others?

Yes

Reviewer Expertise:

emerging infectious diseases, clinical virology, epidemiology/ public health

I confirm that I have read this submission and believe that I have an appropriate level of expertise to confirm that it is of an acceptable scientific standard, however I have significant reservations, as outlined above.

## References

[1] : BCG vaccine protection from severe coronavirus disease 2019 (COVID-19). *Proceedings of the National Academy of Sciences*.2020;117(30) : 10.1073/pnas.2008410117 17720-17726 10.1073/pnas.2008410117 PMC739550232647056

[2] : Viral RNA Load in Mildly Symptomatic and Asymptomatic Children with COVID-19, Seoul, South Korea. *Emerging Infectious Diseases*.2020;26(10) : 10.3201/eid2610.202449 2497-2499 10.3201/eid2610.202449 PMC751074332497001

[3] : A study on infectivity of asymptomatic SARS-CoV-2 carriers. *Respir Med*.169: 10.1016/j.rmed.2020.106026 106026 10.1016/j.rmed.2020.106026 32513410PMC7219423

[4] : Neutralizing Antibody Production in Asymptomatic and Mild COVID-19 Patients, in Comparison with Pneumonic COVID-19 Patients. *J Clin Med*.2020;9(7) : 10.3390/jcm9072268 10.3390/jcm9072268 32708872PMC7408950

# Response

**Published:** 2020-11-09

**Authors:** Philip Machanick

**Affiliations:** Rhodes University, South Africa

Thank you for constructive comments on the paper. Some of the the issues you raise are a consequence of trying to get this out quickly when this issue was fresh (version 1 was published on 4 May 2020). Unfortunately it has taken so long to find reviewers that some issues are out of date. I am happy to update the paper taking your comments into account.

 The asymptomatic *vs*. pre-symptomatic question is a good one. Since I wrote the paper increasing evidence has been published of viral shedding prior to symptoms showing.

 Your points on BCG are also good. As with several of your other points, missing some of this was a consequence of the long delay between submission and review.

 I will work through the other points along with any other reviews received.

 A thought experiment to clarify the role of *R*_0_ and *R _t_*.

*R*_0_ depends on 3 factors (slightly simplifying Delamater et al. 2019):

1. inherent infectiousness of the pathogen
2. how long an infected person remains contagious
3. social mixing

Assume *R*_0_ = 2.5 in a particular society (we must define this societally because of factor 3 – assuming susceptibility is uniform).

 While *R _t_* is a current measure as opposed to *R*_0_, which is a base measure when everyone is susceptible, is it correct to say that NPIs change *R _t_* and not *R*_0_?

 Imagine a particularly NPI is in effect *when patient zero is imported*. Then *R*_0_ will *appear* to be lower than without that NPI, because you have varied factor 3. However: if you wait until 40% of the population is infected and lift all NPIs, you will still expect to see herd immunity at 60% infected (based on *R*_0_ = 2.5). So an NPI fakes the effect of a disease with lower *R*_0_ though the measured effect is seen in *R _t_*. Relax the NPI, and you put yourself onto a later part of the trajectory with a lower fraction susceptible than at the start, but *R _t_* will now climb to the point it would have been without the NPI with that fraction (> 0) susceptible.

 So: I argue that even with NPIs in place, you really need to know *R*_0_, because that determines the inflection point at which cases decline if you relax your NPIs *even if NPIs do not actually change *R *_0_*. Otherwise you get the all-too-typical active-case curve that achieves a neat peak as if you reached herd immunity then takes off again when measures relax.

 There is a lot of confusion about *R*_0_ and *R _t_* so it is good to get these ideas straight. I could add this to the paper if you consider this useful; otherwise it can stay here as a comment.

**Reference**

 Delamater PL, Street EJ, Leslie TF, Yang YT, Jacobsen KH. Complexity of the Basic Reproduction Number (R _0_). Emerg Infect Dis. 2019 Jan;25(1):1-4. doi: 10.3201/eid2501.171901. PMID: 30560777; PMCID: PMC6302597.

# Response

**Published:** 2021-04-09

**Authors:** Philip Machanick

**Affiliations:** Rhodes University, South Africa

1. Define variables in equation 1 below the equation. 
  Done – *E* is the only new variable and is now explicitly rather than implicitly defined.
2. Apart from R _0_, the concept of the effective reproduction number should also be introduced as a much more meaningful figure when NPIs are enforced.
  Done – I explain the role of *R _t_* in determining progress of a pandemic and referred to it as needed.
3. I recall closer to 700 cases on Diamond Princess. Please verify.
  The number is from the reference I cite. I checked and cannot find one that contradicts this.
4. “What is startling about these numbers is how few tested positive. Given the constrained environment of a cruise liner, a higher rate of social mixing would be expected than in normal living conditions.” This is somewhat simplistic. Once the outbreak started, you would expect significant reduction in social interactions and increasing mask usage/ isolation among passengers on board the Diamond Princess, which would lower the R _t_.
  Reworded to reflect this.
5. I do not understand how figure 2 and the equations relate to the rest of the paper. In fact, the first paragraph of the methods appears unrelated to the narrative case study/ review in the rest of the manuscript. 
  I am trying to make the case that there is a phase of a pandemic where exponential growth will overtake health systems. Clarified.
6. I agree with the author on the importance of detecting asymptomatic shedders, but most current estimates of true asymptomatic COVID-19 rates are less than 50% (15 – 30% according to some meta-analyses). This needs to be updated. There is also a need to differentiate between true asymptomatic infection and ‘pre-symptomatic’ cases who are detected before eventually going on to detect symptoms.
  I found meta-analyses supporting 15–20%, which I think establishes your point that the fraction could be significantly lower. I added also the potential confounder of miscounting pre-symptomatic as asymptomatic.
7. Consider citing Escobar LE *et al* ^1^, PNAS as a peer-reviewed reference on the impact of BCG vaccination on severe COVID-19.
  Done, thanks for the reference.
8. BCG vaccination is unlikely to offer sterilizing immunity against COVID-19. Instead, it is mostly being investigated for its effect on ameliorating immune responses to prevent severe COVID-19. Therefore, we can’t rely on BCG vaccination to slow spread of COVID-19 and it is not a replacement for good-practice NPIs. Any country that practices high standards of NPIs, test-and-trace, and isolation-and-quarantine would be able to control COVID-19 irrespective of its BCG coverage. I feel the text is implying that BCG vaccination is under investigation for reducing COVID-19 community burden and this should be modified.
  Done – and added a caution based on hindsight that correlation ≠ causation, with the example of a similar study of ivermectin in Peru that does not appear to be holding up.
9. “Another possible mitigating factor is that existing HIV remedies are among those being investigated for efficacy against COVID-19. However, it would be foolhardy to rely on this as a mitigating factor without solid evidence that it applies widely enough to matter.” May delete this point as lopinavir/ritonavir has been proven to be of no benefit in hospitalized COVID-19 patients in the WHO SOLIDARITY trial.  
  A useful update – a reader who compares versions of the paper and your review will see this history so I am happy to delete this as requested. This illustrates the value of the open review model; the reader can see how our (not only – my) undestanding of the pandemic has evolved since the initial version of the paper.
10. For the points in proposed research, several natural experiments looking at viral shedding in asymptomatic individuals (e.g. Han MS et al, Emerg Infect Dis ^2^) and infectivity of asymptomatic cases (Gao M et al, Respir Med ^3^) have already been described. Characterization of humoral and T-cell responses to COVID-19 have also been done in mild/ asymptomatic cases (Ko JH et al, J Clin Med ^4^). The author may consider updating this section and reviewing the literature thoroughly to identify current research gaps.
  Thank you for the references. I have read the Gao et al. paper and it seemed to me methodologically weak and this is supported by a follow-up letter by Silverman et al. ^5^. However you make a good point that I should review progress in this regard so I have added new references to this section and added the new gap of understanding how vaccines control infectiousness. Han et al. is interesting, thanks. That led me to finding a more recent more authoritative study, Sayampanathan et al. ^6^ I found a good reference on T cell response, Sekine et al ^7^. Thanks for pointing to the need to look into this further.
11. The methods section in the abstract promised an examination of the strategy of using cloth face masks. However, there is no substantive discussion of cloth face masks in the text.
  I removed this and toned down the BCG vaccine claim in the abstract. The main focus of the paper is clarified as emphasising the value of understanding asymptomatic and mild cases – even though the immediate medical emergency with a novel disease is saving the most severe cases.

**References**

 1. Escobar L, Molina-Cruz A, Barillas-Mury C: BCG vaccine protection from severe coronavirus disease 2019 (COVID-19). *Proceedings of the National Academy of Sciences*. 2020; **117** (30): 17720-17726

 2. Han M, Seong M, Kim N, Shin S, et al.: Viral RNA Load in Mildly Symptomatic and Asymptomatic Children with COVID-19, Seoul, South Korea. *Emerging Infectious Diseases*. 2020; **26** (10): 2497-2499

 3. Gao M, Yang L, Chen X, Deng Y, et al.: A study on infectivity of asymptomatic SARS-CoV-2 carriers. *Respir Med*. **169**: 106026

 4. Ko JH, Joo EJ, Park SJ, Baek JY, et al.: Neutralizing Antibody Production in Asymptomatic and Mild COVID-19 Patients, in Comparison with Pneumonic COVID-19 Patients. *J Clin Med*. 2020; **9** (7).

 5. Silverman, E. P., Reddy, R., Ataya, A., & Eagan, C. (2020). Letter to the Editor concerning A study on infectivity of asymptomatic severe acute respiratory syndrome coronavirus 2 (SARS-CoV2) carriers by Gao et al. *Respiratory Medicine*, 106061. Advance online publication. https://doi.org/10.1016/j.rmed.2020.106061

 6. Sayampanathan AA, Heng CS, Pin PH, Pang J, Leong TY, Lee VJ. Infectivity of asymptomatic versus symptomatic COVID-19. *The Lancet*. 2021 Jan 9;397(10269):93-4.

 7. Sekine T, Perez-Potti A, Rivera-Ballesteros O, Strålin K, Gorin JB, Olsson A, Llewellyn-Lacey S, Kamal H, Bogdanovic G, Muschiol S, Wullimann DJ. Robust T cell immunity in convalescent individuals with asymptomatic or mild COVID-19. *Cell*. 2020 Oct 1;183(1):158-68.
